# A Multi-Country Study of Risk and Protective Factors for Emotional and Behavioral Problems Among Early Adolescents

**DOI:** 10.1016/j.jadohealth.2022.05.002

**Published:** 2022-10

**Authors:** Shoshanna L. Fine, Rashelle J. Musci, Judith K. Bass, Effie Chipeta, Eric M. Mafuta, Anggriyani W. Pinandari, Siswanto A. Wilopo, Xiayun Zuo, Robert W. Blum

**Affiliations:** a Department of Population, Family and Reproductive Health, Johns Hopkins Bloomberg School of Public Health, Baltimore, Maryland; b Department of Mental Health, Johns Hopkins Bloomberg School of Public Health, Baltimore, Maryland; c Center for Reproductive Health, College of Medicine, University of Malawi, Blantyre, Malawi; d Department of Epidemiology and Biostatistics, Faculty of Medicine, Kinshasa School of Public Health, University of Kinshasa, Kinshasa, Democratic Republic of Congo; e Center for Reproductive Health, Faculty of Medicine, Public Health, and Nursing, Universitas Gadjah Mada, Yogyakarta, Indonesia; f NHC Key Lab of Reproduction Regulation (Shanghai Institute for Biomedical and Pharmaceutical Technologies), Fudan University, Shanghai, China; g Department of Biostatistics, Epidemiology, and Population Health, Faculty of Medicine, Public Health, and Nursing, Universitas Gadjah Mada, Yogyakarta, Indonesia

**Keywords:** Early adolescents, Psychosocial development, Emotional and behavioral problems, Low- and middle-income countries, Risk and protective factors

## Abstract

**Purpose:**

Early adolescence (ages 10–14) is a critical period for psychosocial development, but few studies have focused on risk and protective factors for emergent psychosocial challenges among youth living in low- and middle-income countries. This study explored the contribution of social environmental factors to patterns of emotional and behavioral problems among early adolescents across four low- and middle-income countries.

**Methods:**

Participants were drawn from the Global Early Adolescent Study, and included 10,437 early adolescents from six low-resource urban settings in the Democratic Republic of Congo, Malawi, Indonesia, and China. Multivariate latent class regression was used to examine the associations between distinct patterns of emotional and behavioral problems and risk and protective factors across the family, peer, school, and neighborhood levels.

**Results:**

Across countries, childhood adversity, peer bullying behaviors, and a perceived lack of school safety were consistently associated with emotional and behavioral problems. With some contextual variability, peer substance use and a perceived lack of neighborhood safety also emerged as significant risk factors. The magnitude of these associations was generally greatest among a subgroup of early adolescents with co-occurring emotional and behavioral problems.

**Discussion:**

The overall consistency of findings across countries is suggestive of the generalizability of risk factors in early adolescence and indicates that interventions bolstering psychosocial adjustment among this age group may have applicability in diverse cross-national settings. Given the significance of peer bullying behaviors and school safety, multicomponent school-based interventions may be an especially applicable approach.

**Implications and Contribution**

This study explores the contributions of social environmental factors to co-occurring psychosocial challenges among early adolescents living in four low- and middle-income countries. The consistency of significant risk factors suggests that interventions targeting psychosocial adjustment among early adolescents may have applicability in diverse cross-national settings.

Early adolescence (ages 10–14) is a critical period for psychosocial development, with the emotional and behavioral problems that commonly emerge during this time elevating the risk of life-long impairment [1,2]. Although there is growing consensus around the importance of intervening during early adolescence in order to lay a foundation for future well-being [3], this period has largely been neglected by researchers, program implementers, and policymakers [4]. Furthermore, despite 90% of the world’s adolescents living in low- and middle-income countries (LMICs), very little research on psychosocial development has been conducted in these settings [1]. Given the heightened vulnerability of early adolescents in low-resource environments [5], such work is essential for shaping interventions that can mitigate risk among disadvantaged youth around the globe.

A critical entry point into such preventive efforts lies in the overlapping social environments that shape adolescent psychosocial development, with potentially modifiable risk and protective factors at the family, peer, school, and neighborhood levels. Within families, exposure to adverse conditions (e.g., abuse and neglect, parental mental illness, economic deprivation) are strongly linked to emotional and behavioral problems throughout the life course [6]. Conversely, positive parenting—including such factors as warmth, communication, authoritativeness, consistent discipline, and monitoring—is a robust protective factor in psychosocial adjustment [7]. Although strong connections with peers can protect adolescents against a range of negative outcomes [8], peer participation in risky health-related behaviors often elevates adolescents’ adoption of these behaviors (e.g., substance use, bullying) [9]. At the school level, connectedness and teacher support are predictive of well-being [10], whereas feeling unsafe is a key determinant of mental health issues [11]. Finally, there is some evidence that neighborhood violence, discrimination, and disadvantage may negatively impact psychosocial adjustment, although findings in this area have been decidedly mixed [12].

Beyond the lack of studies from LMICs, a further limitation of the extant literature is its historical focus on risk and protective factors as they relate to *singular* mental health challenges [13]. This is problematic due to the common co-occurrence of emotional and behavioral problems during early adolescence, with the majority of youth experiencing issues across multiple psychosocial domains [14]. Given this co-occurrence, a focus on individual conditions hampers public health efforts to design and implement multifaceted prevention programs targeting vulnerable youth. In addressing this limitation, a growing number of studies have employed person-centered statistical approaches, such as latent class analysis (LCA), which allow for the identification of subgroups of adolescents who share similar patterns of emotional and behavioral problems [15]. These methods have particular applicability in prevention research, as they can be used to inform targeted responses for those who may be at the highest risk [16]. Although recent investigations have used person-centered approaches to examine risk and protective factors for co-occurring psychosocial challenges among adolescents from a range of diverse contexts [17,18], most have focused exclusively on family- or peer-level factors, precluding their ability to disentangle the relative influence of a broader range of social determinants. Furthermore, no studies have been identified that examine these issues among adolescents across multiple country settings, limiting the generalizability of findings.

The current study attempts to fill such gaps by exploring the contributions of social environmental factors to co-occurring psychosocial challenges among early adolescents living in four LMICs. This study builds on previous research that used multiple-group LCA to identify prototypical patterns (i.e., classes) of emotional and behavioral problems among early adolescents in the Democratic Republic of Congo (DRC), Malawi, Indonesia, and China [19]. In the present analysis, we examine the extent to which risk and protective factors across family, peer, school, and neighborhood environments are associated with class membership. We have chosen to present this separately from the development and testing of latent classes as we feel that the unique methods and substantive findings from the initial analysis warranted a standalone publication.

## Methods

### Study design and sample

Data were drawn from the Global Early Adolescent Study (GEAS), a longitudinal study of risk and protective factors for healthy development among early adolescents living in low-resource urban settings. Participants were sampled from secondary schools in Kinshasa, DRC; Blantyre, Malawi; Semarang, Bandar Lampung, and Denpasar, Indonesia; and Shanghai, China. These countries were chosen from among those participating in the GEAS in order to compare LMICs with diverse cultural, economic, and geographic environments. Baseline data collection was completed by trained data collectors between 2017 and 2018. The majority of questionnaires were self-administered by adolescents via mobile tablets with the exception of DRC, where literacy concerns precluded self-administered surveys. In DRC and Indonesia, primary caregivers were also interviewed in order to provide sociodemographic information. Prior to survey administration, informed consent was obtained from adolescents’ primary caregivers and assent was obtained from adolescents. Study approval was received from Institutional Review Boards (IRBs) of the primary research institution in each participating country as well as the Johns Hopkins Bloomberg School of Public Health IRB. Detailed country-specific study procedures for the GEAS have been described previously [20].

### Measures

#### Latent classes of psychosocial risks

**Figure 1:**
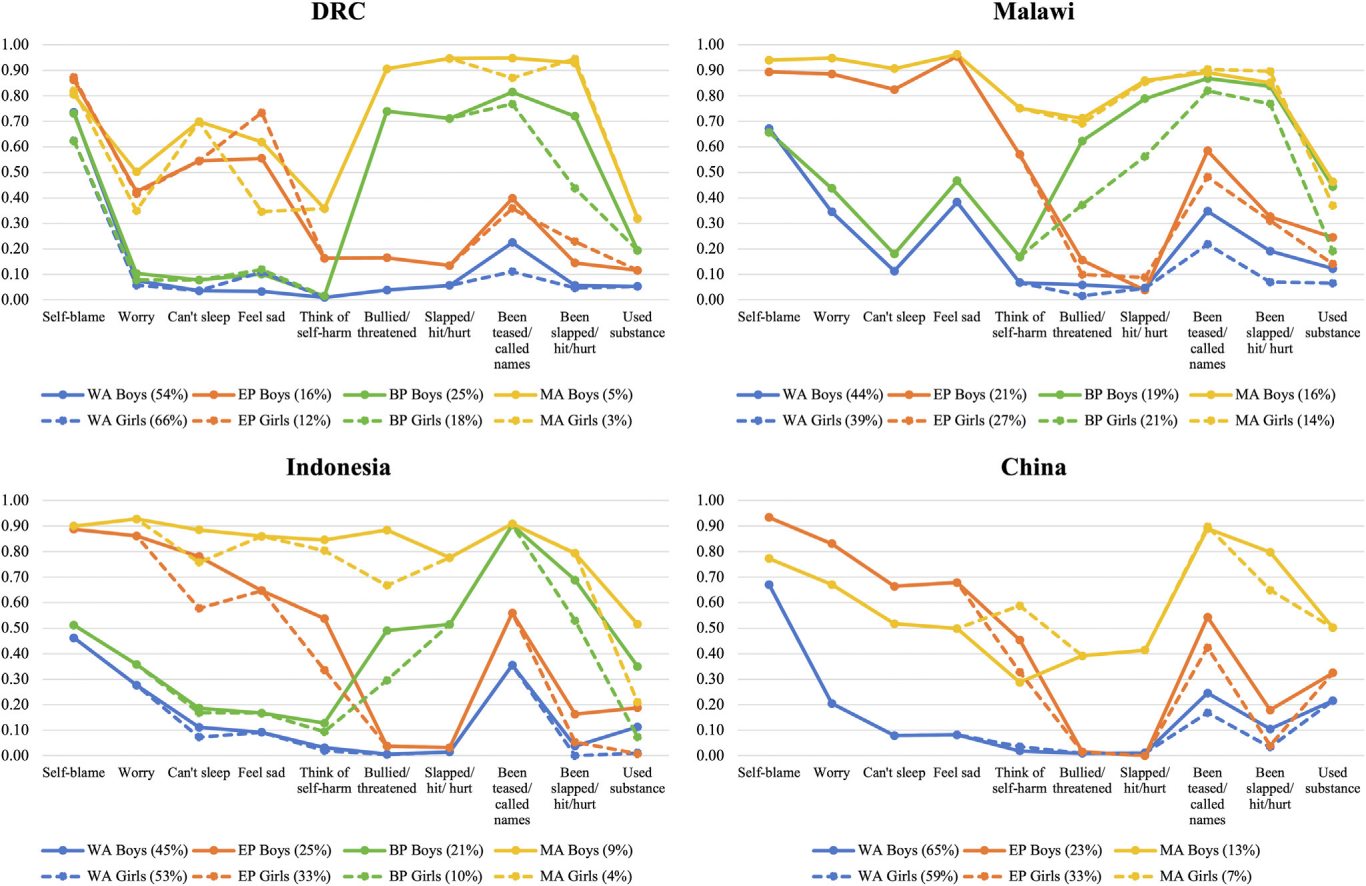
Estimated item-response probabilities for the partially invariant multigroup models in each country. BP = Behavioral Problems; DRC = Democratic Republic of Congo; EP = Emotional Problems; MA = Maladjusted; WA = Well-Adjusted.

Our previous research found that early adolescent patterns of emotional and behavioral problems were best characterized by a four-class latent variable solution in DRC, Malawi, and Indonesia, and a three-class latent variable solution in China. Furthermore, tests of measurement invariance indicated that the nature of these classes differed significantly by sex in each country. As such, partially invariant multigroup models were specified in each country. Parameter estimates for these models are illustrated in Figure 1. Among boys and girls across countries, four general patterns were identified: a *Well-Adjusted* class, with few emotional and behavioral problems (44%–65% boys, 39%–66% girls); an *Emotional Problems* class, with elevated symptoms of depression and anxiety (16%–25% boys, 12%–33% girls); a *Behavioral Problems* class, with increased involvement in aggressive behaviors, peer victimization, and substance use (19%–25% boys, 10%–21% girls; not present in China); and a *Maladjusted* class, with co-occurring emotional and behavioral problems (5%–16% boys, 3%–14% girls) (see Fine et al. for detailed analytic procedures) [19].

#### Psychosocial risk indicators

The latent classes described above relied on 10 indicators related to emotional and behavioral problems. *Emotional* *problems* were measured using five indicators capturing symptoms of depression and anxiety (“I blame myself when things go wrong,” “I worry for no good reason,” “I am so unhappy I can’t sleep at night,” “I feel sad,” “I am so unhappy I think of harming myself”). Adolescents rated how much they agreed with each item using a five-point scale, with response options ranging from “disagree a lot” to “agree a lot.” In order to allow for simultaneous analysis alongside the dichotomous behavioral problem indicators, responses were dichotomized so that a one (1) indicated any agreement and a zero (0) indicated no agreement. *Behavioral* *problems* were measured using five indicators capturing interpersonal aggression and substance use: four that assessed past 6-month experiences of bullying and physical aggression as a victim (“been teased or called names,” “been slapped, hit, or otherwise physically hurt”) or perpetrator (“bullied or threatened,” “slapped, hit, or otherwise physically hurt”), and one that captured lifetime use of one or more substances (alcohol, tobacco, marijuana, and/or illicit drugs). Victimization was considered alongside perpetration given substantial evidence that these experiences are often linked [21] and together may serve as an important marker of psychosocial maladjustment [22]. Across all of the behavioral problem indicators, response options included “yes” or “no.”

#### Risk and protective factors

In total, 10 risk and protective factors across family, peer, school, and neighborhood environments were examined as potential predictors of latent class membership. These risk and protective factors were selected on the basis of their theoretical importance across countries, existing evidence regarding their influence on mental health and well-being, and their consistent availability within the GEAS dataset. *Caregiver connectedness* was assessed with one item: “How comfortable do you feel talking with your primary caregiver about things that worry you?” *Caregiver monitoring* was evaluated with one item: “To what extent does your primary caregiver usually know where you are?” In both cases, responses were dichotomized as “somewhat/very” or “not at all/not very.” *Childhood adversity* was assessed by summing scores from a 13-item Adverse Childhood Experiences (ACEs) measure capturing lifetime experiences of maltreatment (e.g., physical or sexual abuse) and family adversity (e.g., caregiver substance use, economic deprivation) [23]. *Peer socialization* was measured using one item: “During a normal week, how often do you spend time hanging out with your closest friends outside of school?” Responses were dichotomized as “often/very often” or “never/not very often.” *Peer substance use* captured adolescents who reported that “a few,” “most,” or “all” of their friends used tobacco, alcohol, and/or drugs, and *peer bullying* captured those who reported seeing any of their peers bully or threaten someone during the past 6 months. *School* *support* captured adolescents who felt there was an adult at school who really cared about them, whereas *school safety* captured those who indicated feeling unsafe or threatened on the way to school, in the classroom, and/or on school grounds. *Neighborhood cohesion* was assessed by summing scores from four items (α = 0.60–0.90) developed for the GEAS (e.g., “people in my neighborhood look out for and help their neighbors”). Finally, *neighborhood safety* captured adolescents who indicated feeling unsafe or threatened in their neighborhoods.

### Data analysis

A three-step multivariate latent class regression approach was used to examine the associations between latent class membership and risk and protective factors in each country. This approach allows for the addition of covariates into the LCA model through an estimation process that accounts for measurement error due to uncertainty in class classification [24]. The Bolck-Croon-Hagenaars (BCH) three-step analytic method was used to address classification error, as it has been shown to outperform similar approaches [25]. Missing data on the latent class indicators were accounted for through the use of full information maximum likelihood estimation. The rates of missing indicators were no more than 5% for the emotional problem indicators and no more than 11% for the behavioral problem indicators. Missing data on the covariates were addressed with multiple imputation through the *Mplus* program [26]. In general, the rates of missing covariates were no more than 10% across the risk and protective factors. The exceptions to this were the ACEs scale, which had item-level missingness of up to 14% in Indonesia; peer substance use, which had missingness of 18% in Indonesia and 16% in China; and peer bullying, which had missingness of 18% in Indonesia and 19% in China. Standard errors were adjusted for clustering at the school level through the use of sandwich estimators. All multinomial logistic regression models controlled for sociodemographic factors including sex, household size, caregiver marital status, caregiver education, and caregiver employment status. All analyses were performed in *Mplus* version 8.1.6 [27].

## Results

A total of 10,437 early adolescents (ages 10–14) were included across DRC (n = 2,006; 51.5% girls), Malawi (n = 2,016; 49.6% girls), Indonesia (n = 4,657; 53.0% girls), and China (n = 1,758; 48.6% girls). The average age of participants ranged from 11.9 (standard deviation = 1.4) years old in DRC to 12.5 (standard deviation = 1.0) years old in China. Primary caregivers across the three study sites with reported data were relatively well-educated, with the majority having attended secondary school or higher (DRC: 89.1%; Indonesia: 81.7%; China: 82.7%), and most were employed or retired (DRC: 75.6%; Indonesia: 58.0%; China: 85.0%). Sociodemographic characteristics for the sample are presented in Table 1.

**Table 1:** Adolescent sociodemographic characteristics and risk and protective factors

	DRC (n = 2,006)	Malawi (n = 2,016)	Indonesia (n = 4,657)	China (n = 1,758)
Girls, n (%)	1,033 (51.5)	999 (49.6)	2,469 (53.0)	855 (48.6)
Age, M ± SD	11.9 ± 1.4	12.1 ± 1.1	12.2 ± 0.5	12.5 ± 1.0
Household size	7.3 ± 2.6	5.7 ± 1.9	4.8 ± 1.3	3.7 ± 1.1
Primary caregiver’s marital status				
Married/living together	942 (47.0)	-	4,159 (89.3)	1,530 (87.0)
Unmarried/separated/widowed	996 (49.7)	-	272 (5.8)	190 (10.8)
Primary caregiver’s education				
Primary school or less	118 (5.9)	-	631 (13.6)	78 (4.4)
Some or all secondary school	1,045 (52.1)	-	2,309 (49.6)	340 (19.3)
Some or all vocational school or university	742 (37.0)	-	1,493 (32.1)	1,115 (63.4)
Primary caregiver’s employment status				
Employed/retired	1,516 (75.6)	-	2,701 (58.0)	1,495 (85.0)
Unemployed	423 (21.1)	-	1,601 (34.4)	199 (11.3)
Family-level factors				
Comfortable talking to caregiver	1,596 (79.6)	1,542 (76.5)	3,088 (66.3)	1,208 (68.7)
Caregiver knows location	1,588 (79.2)	1,663 (82.5)	3,773 (81.0)	1,584 (90.1)
Adverse childhood experiences	2.0 ± 1.9	2.6 ± 2.8	2.8 ± 2.5	2.4 ± 2.0
Peer-level factors				
Socializes outside of school	1,386 (69.1)	1,367 (67.8)	2,069 (44.4)	229 (13.0)
Peer substance use	248 (12.4)	566 (28.1)	1,348 (29.0)	303 (17.2)
Peer bullying/threatening	1,553 (77.4)	1,347 (66.8)	2,112 (45.4)	416 (23.6)
School-level factors				
Presence of a caring teacher	1,430 (71.3)	1,653 (82.0)	3,384 (72.7)	1,484 (84.4)
Feels unsafe in or around school	339 (16.9)	792 (39.3)	1,442 (31.0)	175 (9.9)
Neighborhood-level factors				
Neighborhood cohesion	7.9 ± 2.6	10.3 ± 2.1	9.4 ± 2.1	8.7 ± 2.9
Feels unsafe in neighborhood	375 (18.7)	226 (11.2)	575 (12.4)	48 (2.7)

In Indonesia and DRC, household size, primary caregiver’s marital status, primary caregiver’s education, and primary caregiver’s employment status are based on caregiver-reported data. In Malawi, primary caregiver’s marital status, education, and employment status are not reported.

DRC = Democratic Republic of Congo; SD = standard deviation.

Parameter estimates from the multinomial logistic regression models in each country are presented in Table 2. Across these results, the *Well-Adjusted* class was used as the reference, as the primary aim of this study is to determine risk and protective factors for psychosocial maladjustment. At the family level, increases in ACEs were consistently associated with elevated likelihood of membership in all of the psychosocial risk classes compared to the *Well-Adjusted* class. Notably, the magnitude of this association was greatest across countries in the *Maladjusted* class (odds ratio [OR] = 1.63–2.12). Caregiver connectedness served as a protective factor only in DRC, where it was associated with decreased odds of being in the *Maladjusted* class (OR = 0.39, *p* = .004). Similarly, caregiver monitoring was associated with reduced likelihood of membership in the *Maladjusted* class in DRC (OR = 0.33, *p* < .001), as well as the *Behavioral Problems* class in Malawi (OR = 0.57, *p* = .045).

**Table 2:** Associations between multilevel risk and protective factors and psychosocial risk classes

	DRC (n = 2,006)	Malawi (n = 2,016)	Indonesia (n = 4,657)	China (n = 1,758)
	OR (95% CI)	OR (95% CI)	OR (95% CI)	OR (95% CI)
*Emotional ProblemsClass*				
Family				
Comfortable talking to caregiver	1.36 (0.83–2.21)	0.93 (0.79–1.08)	1.15 (0.86–1.53)	0.94 (0.60–1.49)
Caregiver knows location	0.84 (0.53–1.33)	0.79 (0.43–1.46)	1.03 (0.82–1.30)	1.23 (0.90–1.69)
Adverse childhood experiences	1.70 (1.47–1.96)∗∗∗	1.31 (1.21–1.42)∗∗∗	1.42 (1.33–1.52)∗∗∗	1.42 (1.18–1.70)∗∗∗
Peer				
Socializes outside of school	0.67 (0.42–1.06)	1.69 (1.46–1.95)∗∗∗	1.28 (1.02–1.62)∗	0.76 (0.35–1.64)
Peer substance use	1.08 (0.51–2.31)	1.80 (1.39–2.33)∗∗∗	1.04 (0.79–1.36)	1.17 (0.71–1.92)
Peer bullying/threatening	3.13 (1.63–6.01)∗∗	1.56 (1.17–2.07)∗∗	1.39 (1.17–1.66)∗∗∗	1.51 (0.98–2.32)
School				
Presence of a caring teacher	1.34 (0.80–2.23)	0.71 (0.42–1.18)	1.11 (0.82–1.50)	0.71 (0.27–1.88)
Feels unsafe in or around school	2.00 (1.16–3.46)∗	1.38 (1.03–1.84)∗	1.61 (1.27–2.05)∗∗∗	1.68 (1.43–1.98)∗∗∗
Neighborhood				
Neighborhood cohesion	1.02 (0.94–1.11)	1.06 (0.92–1.21)	1.05 (1.00–1.11)∗	0.96 (0.88–1.04)
Feels unsafe in neighborhood	0.72 (0.43–1.20)	1.29 (0.93–1.80)	1.63 (1.21–2.19)∗∗∗	2.13 (1.54–2.95)∗∗∗
*Behavioral ProblemsClass*				
Family				
Comfortable talking to caregiver	0.95 (0.67–1.35)	1.02 (0.64–1.65)	1.20 (0.93–1.55)	-
Caregiver knows location	0.80 (0.59–1.10)	0.57 (0.33–0.99)∗	0.73 (0.50–1.06)	-
Adverse childhood experiences	1.29 (1.17–1.43)∗∗∗	1.35 (1.20–1.53)∗∗∗	1.44 (1.37–1.52)∗∗∗	-
Peer				
Socializes outside of school	1.56 (1.09–2.24)∗	1.23 (0.86–1.78)	1.23 (0.90–1.67)	-
Peer substance use	1.28 (0.80–2.06)	1.90 (1.35–2.68)∗∗∗	1.41 (1.03–1.93)∗	-
Peer bullying/threatening	13.67 (5.66–33.02)∗∗∗	6.61 (3.16–13.82)∗∗∗	4.67 (3.10–7.03)∗∗∗	-
School				
Presence of a caring teacher	0.91 (0.65–1.29)	1.14 (0.65–1.99)	0.92 (0.71–1.20)	-
Feels unsafe in or around school	2.23 (1.50–3.31)∗∗∗	2.06 (1.80–2.37)∗∗∗	2.13 (1.72–2.63)∗∗∗	-
Neighborhood				
Neighborhood cohesion	0.97 (0.90–1.03)	1.00 (0.90–1.11)	0.93 (0.87–0.99)∗	-
Feels unsafe in neighborhood	1.54 (1.06–2.24)∗	1.58 (1.21–2.06)∗∗	1.70 (1.20–2.40)∗∗	-
*MaladjustedClass*				
Family				
Comfortable talking to caregiver	0.39 (0.21–0.74)∗∗	1.01 (0.68–1.50)	1.14 (0.74–1.76)	0.53 (0.26–1.07)
Caregiver knows location	0.33 (0.19–0.60)∗∗∗	0.87 (0.39–1.94)	1.04 (0.63–1.71)	0.99 (0.81–1.21)
Adverse childhood experiences	2.12 (1.71–2.64)∗∗∗	1.63 (1.52–1.76)∗∗∗	1.97 (1.81–2.14)∗∗∗	1.76 (1.44–2.16)∗∗∗
Peer				
Socializes outside of school	0.89 (0.39–2.02)	1.52 (1.02–2.27)∗	1.69 (1.14–2.52)∗	1.00 (0.42–2.37)
Peer substance use	2.76 (1.22–6.24)∗	3.06 (1.92–4.87)∗∗∗	1.35 (0.86–2.12)	2.69 (1.77–4.09)∗∗∗
Peer bullying/threatening	-	4.83 (2.84–8.21)∗∗∗	10.78 (4.36–26.67)∗∗∗	3.96 (2.17–7.22)∗∗∗
School				
Presence of a caring teacher	1.42 (0.55–3.67)	0.86 (0.43–1.71)	0.80 (0.48–1.33)	0.41 (0.16–1.09)
Feels unsafe in or around school	2.94 (1.38–6.29)∗∗	2.24 (1.69–2.95)∗∗∗	2.51 (1.56–4.04)∗∗∗	2.40 (1.38–4.19)∗∗
Neighborhood				
Neighborhood cohesion	0.95 (0.81–1.12)	1.05 (0.92–1.20)	1.04 (0.93–1.17)	0.99 (0.94–1.04)
Feels unsafe in neighborhood	1.14 (0.57–2.29)	2.69 (1.50–4.85)∗∗∗	1.73 (1.25–2.39)∗∗	1.35 (0.22–8.23)

*Well-Adjusted* class is the reference class. All models control for sex, household size, primary caregiver’s marital status, primary caregiver’s education, and primary caregiver’s employment status where possible.

CI = confidence interval; DRC = Democratic Republic of Congo; OR = odds ratio.

*∗p* < .05.

*∗∗p* < .01.

*∗∗∗p* < .001.

At the peer level, bullying was associated with significantly increased odds of membership in all of the psychosocial risk classes across countries. The magnitude of these relationships was greatest in the *Behavioral Problems* and *Maladjusted* classes, with those who reported witnessing peers bullying or threatening others around 4–14 times as likely to be in these classes compared to the *Well-Adjusted* class. With a few exceptions, peer substance use was another important risk factor for membership in the *Behavioral Problems* and *Maladjusted* classes (OR = 1.41–3.06); for the *Emotional Problems* class, however, it was only significant in Malawi (OR = 1.80, *p* < .001). Interestingly, peer socialization outside of school increased the odds of membership in both the *Emotional Problems* and *Maladjusted* classes in Malawi and Indonesia (OR = 1.28–1.69), and the *Behavioral Problems* class in DRC (OR = 1.56, *p* = .02).

A lack of safety in school and neighborhood environments emerged as central risk factors across countries. Those who reported feeling unsafe in or around school were consistently more likely to be in all of the psychosocial risk classes, with the strongest associations in the *Maladjusted* class (OR = 2.24–2.94). Feeling unsafe in the neighborhood demonstrated more contextual variability: it increased likelihood of membership in the *Emotional Problems* class in Indonesia (OR = 1.63, *p* < .001) and China (OR = 2.13, *p* < .001), the *Behavioral Problems* class across countries (OR = 1.54–1.70), and the *Maladjusted* class in Malawi (OR = 2.69, *p* < .001) and Indonesia (OR = 1.73, *p* = .001). In terms of protective factors, school support did not reduce the odds of membership in any of the psychosocial risk classes, and neighborhood cohesion was only significant in Indonesia, where it slightly increased the likelihood of being in the *Emotional Problems* class (OR = 1.05, *p* = .03) and slightly reduced the likelihood of being in the *Behavioral Problems* class (OR = 0.93, *p* = .02).

## Discussion

The current study assessed the unique contributions of social environmental factors to psychosocial challenges among early adolescents living in DRC, Malawi, Indonesia, and China. Building on prior research, we explored the extent to which risk and protective factors across the family, peer, school, and neighborhood levels were associated with latent classes of emotional and behavioral problems. Across countries, ACEs emerged as a consistently significant risk factor for psychosocial maladjustment. Although this is not surprising given the well-documented associations between childhood adversity and subsequent mental health and psychosocial problems [6], it contributes to an emerging body of research focused on the detrimental developmental consequences of ACEs in LMICs [23]. Researchers have suggested that childhood adversity may present a particular challenge in low-resource settings due to the compounding effects of chronic poverty, widespread violence, and systemic limitations [28]. In the context of the current study, this hypothesis is strengthened by the cross-national generalizability of the associations between childhood adversity and adolescent emotional, behavioral, and co-occurring problems. It is also noteworthy that the magnitude of this association was greatest in the *Maladjusted* class compared to the *Emotional Problems* and *Behavioral Problems* classes. Given substantial evidence regarding the dose-response relationship between exposure to ACEs and life course mental health problems [6], this suggests that youth who fall within the *Maladjusted* subgroup may be particularly vulnerable to emergent psychopathology.

These findings also affirm the critical role that peer influence plays in adolescent psychosocial development across diverse contexts. Peer bullying behaviors were robustly linked to the manifestation of behavioral problems with or without co-occurring emotional problems, and these associations were echoed, albeit not as strongly or consistently, for those reporting peer substance use. Again, this is not unexpected: a substantial body of literature has emphasized that affiliation with deviant peers is correlated with a range of risky health-related behaviors in adolescence [9]. In the case of peer bullying behaviors, however, the strength of the associations within the *Maladjusted* and *Behavioral Problems* classes speaks to the ubiquity of witnessing, perpetrating, and experiencing bullying within certain subgroups. Increasingly, researchers have recognized bullying as a complex social phenomenon in which perpetrators and victims are embedded within social contexts that can either deter or reinforce these behaviors [29]. Our findings speak to the cross-cultural applicability of this construct, and support the assertion that interventions targeting these behaviors must take social contexts into account in order to maximize their impact [30].

School safety emerged as another salient point of intervention across study countries, with adolescents who reported feeling unsafe in or around school significantly more likely to have psychosocial problems across one or more domains. This is consistent with a growing body of evidence, largely drawn from high-income countries, which suggests that perceived school safety can profoundly influence adolescent well-being [11]. Although school safety is a somewhat nebulous construct, researchers generally agree that it is strongly related to the pervasiveness of interpersonal violence within school contexts [31]. This reinforces the necessity of addressing bullying within schools, as this may act on adolescent well-being by both reducing bullying experiences and increasing feelings of safety. Beyond violence, however, it has been suggested that perceived safety is driven by additional school climate–related factors, including a strong sense of community, teacher support, fair and consistent disciplinary practices, and orderliness [[31], [32], [33]]. This suggests the need for comprehensive interventions targeting both individual behaviors and school-wide practices in order to create safe and supportive learning environments [34].

Although the consistency of influential risk factors across countries is striking, equally notable is the overall lack of significant protective factors. In particular, despite our expectation that family-level protective factors would strengthen adolescent psychosocial adjustment [7], neither caregiver connectedness nor monitoring emerged as protective factors in the majority of countries. The greatest exception to this was in DRC, where both factors decreased the likelihood of membership in the *Maladjusted* class; this relationship did not hold, however, for the *Emotional Problems* or *Behavioral Problems* classes. This overall lack of significance may relate to the shifting importance of peer environments relative to family environments among this age group. Adolescence is characterized by an increased desire for autonomous decision-making, which is facilitated by a social reorientation toward peers [2]. As such, while parenting practices remain important during this period [35], peer influence may ultimately be more dominant, thereby rendering parental factors less significant when held next to broader social environmental factors.

Together, these findings speak to the potential of using multicomponent school-based interventions to bolster psychosocial adjustment among adolescents living in low-resource settings. In particular, the key risk factors identified above suggest an approach in which individual strategies focus on vulnerable youth with co-occurring emotional and behavioral problems, classroom initiatives incorporate violence prevention curricula, and school-wide policies aim to increase safety. This aligns with the World Health Organization’s Health Promoting Schools framework, a holistic model which emphasizes the need to target individuals, classrooms, and whole schools in order to promote health and prevent illness among students [36]. Systematic reviews have suggested that such integrated approaches may be more effective in influencing adolescent psychosocial development than those focusing purely on one strategy [37]. Notably, while few studies of multicomponent school-based interventions have been carried out in LMICs, a recent trial conducted in secondary schools in India found that this approach had substantial impacts on adolescent health and well-being, including reductions in depressive symptoms and bullying behaviors [38].

These findings must be interpreted in light of several limitations. First, the cross-sectional nature of these data precludes statements about causality or temporality in the relationship between social environmental factors and latent class membership. Second, all data were assessed by adolescent self-report and are thus subject to social desirability bias, although the use of computer-assisted self-interview in many of the countries may have helped to mitigate this issue [39]. Third, with the exception of childhood adversity and neighborhood cohesion, the measurement of risk and protective factors relied on single dichotomized items rather than validated scales. It is possible that this measurement limitation led to spurious conclusions about the role of certain factors. For instance, while we found that peer socialization acted as a risk rather than a protective factor in several countries, given its operationalization as time spent with friends outside of school, it is plausible that this covariate captured unsupervised social activities rather than peer connectedness. Indeed, prior research among youth in the United States has found correlations between increased peer activity in the evening and a range of behavioral problems [18]. Finally, it has been suggested that multiple imputation may be inappropriate in a latent class analytic framework due to the theoretical incompatibility between multiple imputation, which assumes a single underlying population, and LCA, which assumes multiple latent subgroups within a population [40].

These limitations notwithstanding, the current study has several important strengths, such as its inclusion of early adolescents from four LMICs, its use of a person-centered analytic approach to examine co-occurring psychosocial challenges, and its simultaneous examination of risk and protective factors across multiple social environmental domains. Across countries, we found a number of factors which were consistently and robustly associated with emotional and behavioral problems, including childhood adversity, peer bullying behaviors, and a perceived lack of school safety. This consistency is suggestive of the generalizability of risk factors in early adolescence, and indicates that interventions targeting psychosocial adjustment among this age group may have applicability in diverse cross-national settings. In addition, the patterns of association across latent classes point to especially heightened vulnerability among a subgroup of adolescents with co-occurring emotional and behavioral problems. Given resource limitations in many LMICs, this information can be used to guide decision-making around which adolescent populations to prioritize through interventions.
